# The Convallis learning rule for unsupervised learning in spiking neuronal networks

**DOI:** 10.1186/1471-2202-14-S1-P426

**Published:** 2013-07-08

**Authors:** Pierre Yger, KD Harris

**Affiliations:** 1 Institute of Neurology, Department of Neuroscience, Physiology and Pharmacology, University College London, London, UK

## 

Learning is often considered to be governed by reinforcement, causing animals to increase the probability of performing rewarded behaviours, and decrease the probability of behaviours leading to harmful outcomes. However, not all learning is governed by reward. Mere exposure to a novel environment or set of sensory stimuli, unpaired with any behaviour or reinforcement, leads to perceptual learning that allows the animal to more readily form behavioural associations with these stimuli, should the need later arise.

In statistics and machine learning, the problem of forming representations of a data set without any explicit training is called "unsupervised learning". Artificial neural networks that perform unsupervised learning have been described, primarily for the case of firing rate neurons. Many of these learning rules allow the networks to perform analyses equivalent to standard statistical techniques. For example, a variant of the Bienenstock, Cooper and Monro (BCM) theory of plasticity allows neurons to implement projection pursuit, a statistical technique for searching for non-Gaussian projections of input data. Here we describe a synaptic rule for unsupervised learning in spiking neurons. The rule is derived as gradient optimization of the time integral of a nonlinear function of membrane potential. This function is shaped like a valley, leading to a preference for membrane potentials near rest or spike threshold, but avoiding intermediate-level potentials (the name "Convallis" comes from the Latin for valley). To avoid saturation, the rule is stabilized by a homeostatic mechanism that enforces a constant firing rate using a PI controller that scales synaptic weights. This combination causes neurons to develop a skewed, non-Gaussian distribution of membrane potentials analogously to the projection pursuit algorithm.

Using the TIDIGITS database of spoken digit utterances, we show the rule allows a recurrent network of spiking excitatory and inhibitory neurons to develop selective representations of these digits. Applying a linear classifier downstream of the recurrent network shows the Convallis rule allows substantially better readout than a purely random network ("liquid-state machine"), the homeostatic rule alone, or various other plasticity rules for spiking neurons. Applying the rule to simulations of in vitro plasticity paradigms, we find that it reproduces several published results including STDP, although STDP alone cannot produce similar performance in speech recognition. We suggest that ability to perform real-world information processing tasks provides a useful way to constrain theories of synaptic plasticity.

